# Supplementary material for: DEPTH2 score was associated with cell proliferation and immune cell infiltrations but not with systemic treatment response in breast cancer
Source: Sci Rep. 2025 Nov 26;15:42225. doi: 10.1038/s41598-025-26379-1 (PMC12657884; doi:10.1038/s41598-025-26379-1)

| **Supplementary Table S1. Clinicopathological characteristics of the included cohorts.** | | | | | | | | | |
| --- | --- | --- | --- | --- | --- | --- | --- | --- | --- |
| **Cohort Name** | **Author** | **Year** | **Sample Size** | **Platform** | **Median Age** | **ER+/HER2– (%)** | **HER2+ (%)** | **TNBC (%)** | **Treatment Regimen** |
| TCGA | Cancer Genome Atlas Network | 2012 | 1077 | Illumina HiSeq | 58 | ~70 | ~20 | ~20 | Standard-of-care |
| METABRIC | Curtis et al. | 2012 | 1904 | Illumina HT-12 microarray | 62 | 72 | 12 | 16 | Standard-of-care |
| SCAN-B | Brueffer et al. | 2018 | 3069 | Illumina HiSeq | 64 | ~75 | ~20 | ~10 | Standard-of-care |
| GSE87455 (PROMIX) | Kimbung et al. | 2018 | 275 | Illumina HT-12 microarray | 50 (mean) | 80 | 0 | 20 | Epirubicin + docetaxel ± bevacizumab |
| GSE28844 | Vera-Ramirez et al. | 2013 | 61 | Affymetrix HG-U133 Plus 2.0 microarray | 54 | ~70 | ~20 | ~30 | Anthracycline + taxane; HER2+ received trastuzumab |
| GSE21974 | Stickeler et al. | 2011 | 57 | Agilent 4×44K microarray | 50 | 47 | 22 | 28 | Epirubicin + cyclophosphamide + docetaxel |
| GSE180280 | Parkes et al. | 2022 | 57 | Illumina NextSeq 500 | 47 | 59 | 35 | 28 | FEC ± docetaxel ± trastuzumab/pertuzumab |
| GSE20194 | Popovici et al. | 2010 | 278 | Affymetrix U133A microarray | 51 | 62 | 26 | 12 | Paclitaxel + 5-FU, doxorubicin, cyclophosphamide |
| GSE25066 | Hatzis et al. | 2011 | 508 | Affymetrix U133A microarray | 49 | 58 | 6 | 36 | Taxane, Anthracycline |
| GSE163882 | Chen et al. | 2022 | 222 | Illumina NextSeq 500 | 54 | 31 | 28 | 41 | Taxane based therapy |
| **Abbreviation: ER+**: Estrogen receptor positive, **HER2+**: Human epidermal growth factor receptor 2 positive, **TNBC**: Triple-negative breast cancer, **FEC**: 5-FU + Epirubicin + Cyclophosphamide. | | | | | | | | | |

**Supplementary Figure Legends**

**Supplementary Figure S1.** Disease free survival outcomes in breast cancer patients by DEPTH2 high and low groups. The Kaplan-Meier survival plots comparing tumors with high (red lines) and low (blue lines) DEPTH2 score in TCGA and METABRIC cohorts.

**Supplementary Figure S1.**


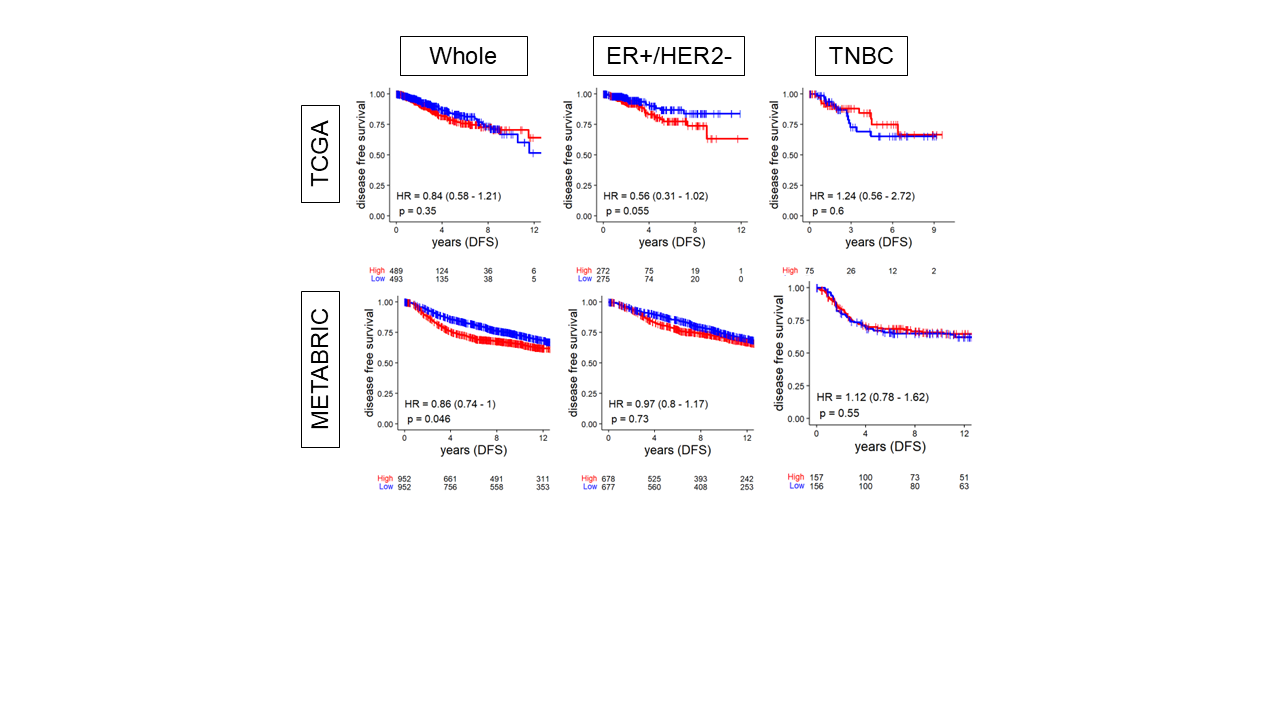


**Supplementary Figure S2**. Association of DEPTH2 score with the Nottingham histological grade by breast cancer subtypes.

**Supplementary Figure S2.**


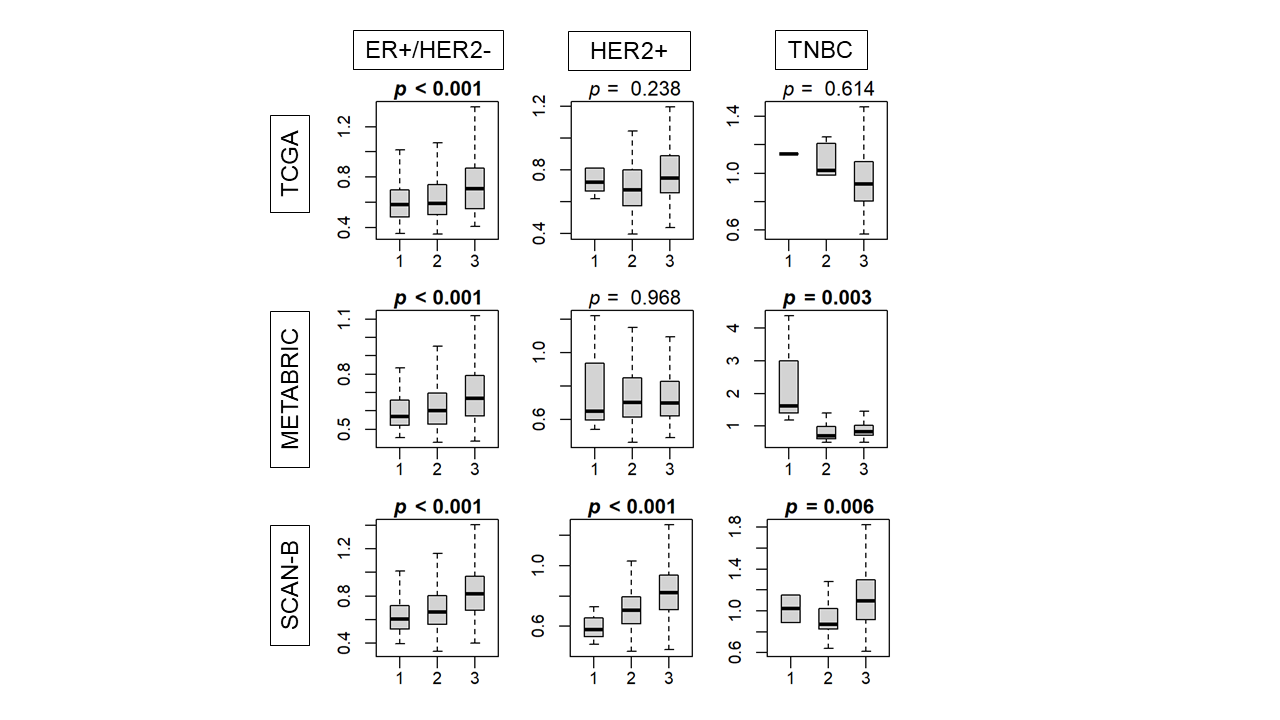


**Supplementary Figure S3**. Association of chemotherapy treatment and its response in breast cancer patients by DEPTH2 high and low groups. Relationship between ITGH score and pathological complete response (pCR) and residual disease (RD) in “whole” cohorts from Popovici et al., Hatzis et al., and Chen et al.

**Supplementary Figure S3.**


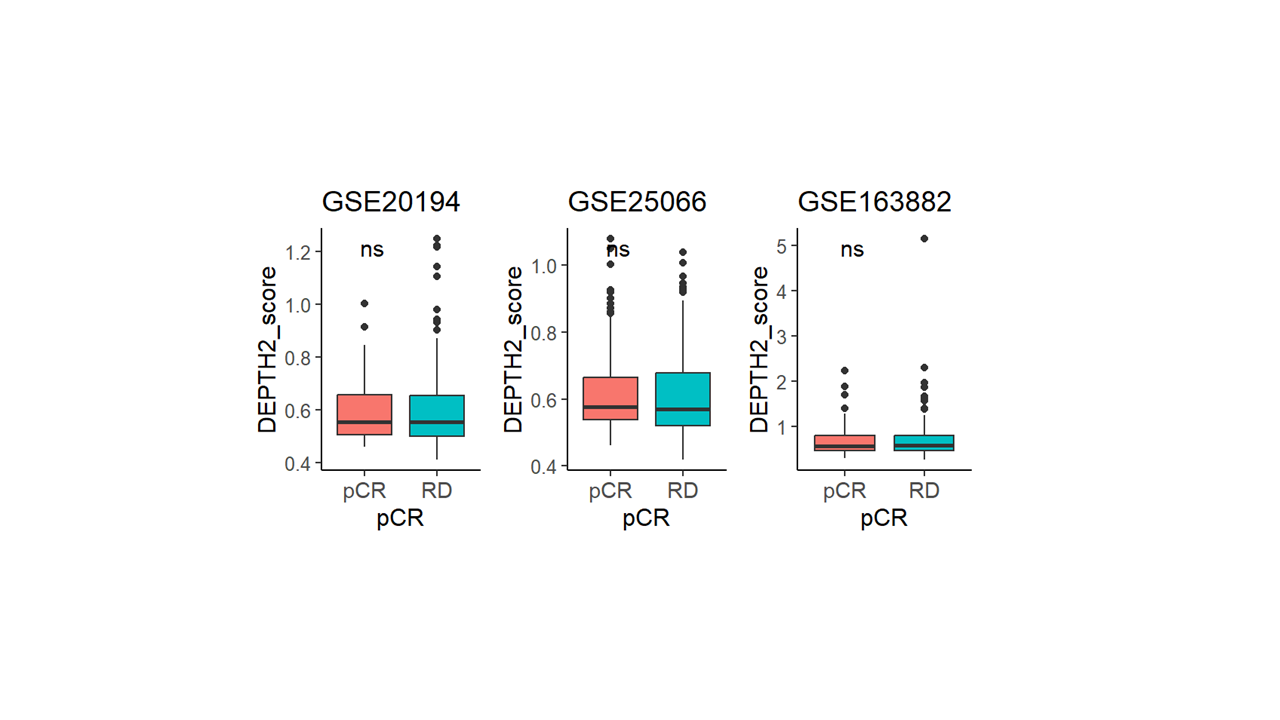

Supplement: Supplementary file 1 — Supplementary Material 1 [file 41598_2025_26379_MOESM1_ESM.docx]
